# Hip Lift Transfer Assistive System for Reducing Burden on Caregiver’s Waist

**DOI:** 10.3390/s21227548

**Published:** 2021-11-13

**Authors:** Jiang Wu, Motoki Shino

**Affiliations:** Department of Human and Engineered Environmental Studies, Graduate School of Frontier Sciences, The University of Tokyo, Kashiwa 277-8563, Japan; motoki@k.u-tokyo.ac.jp

**Keywords:** transfer assistive system, motion analysis, caregivers’ lumbar burden, QOL of elderly

## Abstract

In Japan, the aging population is expected to increase the number of elderly people in the future. The purpose of this study was to develop a hip lift transfer assistive system to improve the QOL of elderly living and to prevent back pain for caregivers. We extracted the impediment factor and the necessary scene for the assistance, decided on the transfer process from the wheelchair in the toilet, and considered the reduction method of the burden based on the quantitative evaluation of the caregiver’s lumbar burden and developed the device. Then we proposed the algorithm of the system by grasping the behavior and lumbar burden characteristics at the time of using the hip lift transfer assistive system by the developed device in which the proposed support algorithm of standing seating assistance operation is implemented in the actual use environment. Through the assistive movement evaluation experiment and the actual operation in the toilet, we have verified that the use of this device can reduce the caregivers’ lumbar burden below the standard value (3400 N) and have proved the effectiveness of the proposed transfer assistive system.

## 1. Introduction

### 1.1. Background

The aging rate has reached 28.1% [1] in Japanese society. Not only Japan, but the global aging trend is also deepening. Based on the prediction of the United Nations, by 2050, one sixth of the population will be over 65 years old. More detailed data suggest that the proportion of the world’s population aged 65 and over will rise from 9% to 16% [2]. It is expected that the number of elderly people in elderly facilities will continue to increase in the future.

Looking at the caregivers in welfare facilities for the elderly, the disaster rate of low back pain is very high. It is reported that 80% of caregivers have a history of low back pain at work and many back pain problems have been reported as issues that need to be solved in order to improve the working environment of caregivers [3,4]. Caregivers carry out heavy work such as lifting the care recipients (the elderly) repeatedly, there are many bending postures and twisting movements, and it is difficult to obtain spontaneous rest, which will also aggravate the mental burden [5]. According to a report by the Ministry of Health, Labour and Welfare (MHLW), 70% of the causes of lower back pain among caregivers are movements (from wheelchair to bed, from wheelchair to toilet, etc.) that support the elderly in transfer [6]. Therefore, it is an important factor in improving the working environment of caregivers [7].

The transfer is a necessary action in communicating, moving, and defecating with others, which is very important to improve the QOL of the elderly. There are two reasons why elderly people should excrete using the toilet instead of diapers. First of all, a sitting posture is more suitable for defecation and improves constipation. Second, better posture may lead to activation of the autonomic nervous system by defecation reflex. However, as mentioned above, transfer assistance is the action with the highest risk of lower back pain in nursing actions. Therefore, in the Countermeasures for the prevention of lower back pain in 2014, in principle, for the actions requiring full care in transfer assistance, they do not need to be picked up manually, but to use the transfer assistive devices [6].

Although various methods have been developed in the research of transfer assistive devices, the Health and Welfare Information Association generally recommends the use of transfer boards and lifts [8]. These recommendations are used separately according to lower limb function of the elderly. The lifts cover the body of the elderly with a cloth called a lifting ring. It is recommended for the elderly who can’t maintain a sitting posture with a lifting method similar to the lifting appliance. The transfer boards are methods to make the buttocks slip by bridging the transfer source and transfer destination. Although it is difficult to stand, it is recommended for the elderly who can maintain the sitting posture and use wrist strength to get their hips out of the seat. In actual use, the effect of reducing the burden on the waist has also been verified [9]. Both can prevent the load concentration of obstacle parts other than the hip, so the adaptation to the disabled elderly has also been fully considered. However, since both transfers operate under the condition that the hips are covered, it is difficult to adapt to the action that the hips need to leave the seat or the transfer (wearing and taking off clothes in the toilet, etc.)

### 1.2. Related Works

Focusing on the transfer movement to the toilet, we investigated transfer assistive devices that can assist lifting the hip out of the seat. It is roughly divided into caregiver assistive type and care recipient assistive type. The caregiver assistive type is a method of wearing an exoskeleton robot suit or a strap type muscle strength assistance suit on the caregiver [10,11,12,13]. The methods for the buttocks to leave the seat in the care recipient assistive type include a device for leaning forward with the upper limbs or upper body to make the buttocks leave the seat, and an auxiliary lift for assisting the elderly to stand under the condition of fixing the knee through the armrest on the armpit and back [14,15,16,17,18,19].

In the toilet, the caregiver needs to disassemble the diaper, deal with the excreta, put on and take off the clothes and other actions for the nursing elderly. Therefore, the movement of the trunk and upper limbs requires degrees of freedom. In addition, the elderly with low lower limb ability need to prevent knee fracture, so a simple caregiver transfer assistive device is not enough. In addition, HAL [10,13] which can assist the whole-body movement, is not suitable for the elderly facilities requiring rapid movement because it takes time to put on and take off. On the other hand, because the elderly can lift their buttocks out of the bed by themselves, the standing auxiliary lift and the forward leaning transfer assistive device can also achieve the freedom of movement for caregivers. Therefore, in the toilet space, the standing auxiliary lift and the forward leaning transfer assistive device are suitable.

Many types of transfer assistive devices have been developed for people. For a literature review [20] related to person transfer assist systems, most of the articles introduced ceiling and floor-mounted lifts, but also pointed out the focus of caregiver injury risk. Few articles focused on the impact on people. R. Hari Krishnan has developed a robotic self-transfer device [21] that allows wheelchair users to transfer themselves from the wheelchair to the toilet. However, there is no consideration for elderly users, it is very important for the elderly to have caregivers around when using automatic devices. The Bending Non-Demand Return (BNDR) weight transfer device (WTD) [22] studied by Brent L. Ulrey showed a significant reduction in lumbar spine erector muscle activity when worn in the bending position. The device may prove to be an effective intervention for workers who often use the bending posture. However, for caregivers who help the elderly with transfer assistance, lumbar bending posture is inevitable. Eline R. Blaauw compared the new robot assisted transfer device (strong arm) with the clinical care standard (Hoyer advance) [23] and measured the muscle activity of caregivers when using both. In order to reduce the injury of caregivers and wheelchair users in the process of transfer, a more ergonomic transportation method was provided. However, the factor that wheelchair users are often elderly is not taken into account. When the elderly use the device, the caregivers need to hold the body to reduce psychological anxiety, the elderly can’t fix their knees, and some functional disorders of their upper limbs also need to be considered.

This study focused on a hip lift transfer assistive system that considered the transfer of the elderly from the bedroom to the toilet, while ensuring that the burden on the waist of caregivers does not exceed the standard value. We measured the actual transfer assistive movements, determined the movement outline of the device, and grasped its necessity and function. In addition, it is also necessary to fully consider the actual movement characteristics of the elderly (anxiety when using devices, general weakness, partial dysfunction of upper limbs, inability to self-fix knees, etc.) to fit the practical application of facilities for the elderly on site.

### 1.3. Positioning of This Study

According to the MHLW documents regarding the elderly living in elderly facilities [24,25], 64% of the elderly are paralyzed and 81% of the elderly are constricted. In the case of upper limb paralysis, there are 30% left and right, 35% shoulder contracture and 29% elbow contracture. It has been reported that the risk for the elderly to have diseases on the upper limbs and upper torso is very high. In addition, because these functional disorders are less related to the sitting retention function, even if there are these obstacles, they also need to be transferred to the toilet. Therefore, this study will not rely on upper limb diseases as a necessary condition for device development. In addition, in the care of the elderly with upper limb dysfunction, there are risks such as obstacle deterioration and fracture in the load and compression of the obstacle parts, so it is necessary to reduce the load of the affected parts of the elderly.

Although the existing transfer assistive device is possible to promote the use through publicity or education, considering that there are problems in the operation of the device during the transfer to the toilet, this study takes the transfer assistance to the toilet as the subject. In addition, the main reasons that hinder the introduction of assistive device in toilet for the elderly need to be considered as follows.

- correspondence of elderly people with upper limb dysfunction
- device structures that can be used in toilet space
- ease of operation
- reduction of labor and time during use

Therefore, the objective of this study is to develop a transfer assistive device that can be applied into the toilet of the elderly facilities, which does not depend on the obstacles of the upper limbs and trunk of the elderly being cared for, and can reduce the burden on the waist of the caregiver and make the hips of the elderly leave the seat.

Specifically, Section 2 introduces the development requirements to adapt to the conditions of elderly care facilities, through the investigation into the elderly facilities, the transfer situation to the toilet, and through the pre-experiments to verify the time, burden, and knee fixation of the transfer movement, so to obtain specific data to complete the design requirements. The functions, specifications, and structures of the device are introduced in Section 3, the movement evaluation using the device and the application in the actual scene are verified in Section 4, and the conclusions are shown in Section 5.

## 2. Development Requirements Adapted to Elderly Facilities Conditions

We investigated the status and opinions of caregivers on the introduction of transfer assistive devices through the visit to the actual elderly facilities, the current situation of transfer in the toilet, and use of the motion capture system to analyze the actual caregiving action to quantitatively grasp the transfer behavior of the caregiver, soto sort out the specific requirements of the transfer assistive device to be developed.

### 2.1. Visit Investigation to the Elderly Facilities

As a prerequisite for device development, we observed and heard the environment and specific work contents of the elderly facilities. We summarized the caregivers’ awareness of transfer, the measures to deal with lower back pain, the safety of the elderly, and then summarized the extraction results of the obstacles to the introduction of transfer assistive device and the requirements for the transfer assistive device.

In detail, we visited nine special nursing homes for the elderly in Tokyo and investigated the current situation of transfer in the toilet. Only two facilities (B1, C1) visited after investigating the devices they were using in advance. Other facilities were randomly selected and visited after obtaining the approval to visit the facilities. As a form of business, we record the average level of care required by residents, the number of residents, the number of day staff, and the number of victims of lower back pain in Table 1. The method adopted to obtain the data was a direct face-to-face interview because the reasons for the selection criteria and impression of transfer assistive devices are unknown.

The number of transfer movements per day varied according to whether the elderly could transfer independently or not, at least eight times. These special nursing homes surveyed need a high degree of care, with an average of about four. Although no accurate value is obtained, few elderly people can transfer independently, and almost all elderly need transfer assistance. In addition, for one caregiver, the ratio of the elderly to be cared for every day is about eight on average. Even if the shift is included, at least 30 people should be transferred during the day. In addition, for the transfer before breakfast, one caregiver on duty should transfer about 15 to 20 people.

In the facilities A1 to A7, some facilities did not know the specific number of those suffering from lower back pain amongst the caregivers. In addition, in 20% to 30% of the facilities, there were many caregivers with mild low back pain despite there were few caregivers with severe low back pain. This specific figure has not been determined. Especially in A6 facilities, the incidence rate of lower back pain of caregivers is 80%, and half of these cases of lower back pain cause obstacles to work. Even in this case, the answer to the transfer awareness was “caregivers are not troubled, find ways to solve and minimize labor”.

Through this survey, we got the result that although the facilities and caregivers were aware of the problem of low back pain and had a transfer board, or other solutions, they gave up using them because they were very troublesome. Therefore, the design of a transfer assistive device that can prevent or reduce caregivers’ low back pain as well as being easy to use has attracted much attention.

### 2.2. Current Status of Transferring in the Toilet

**Figure 1:**
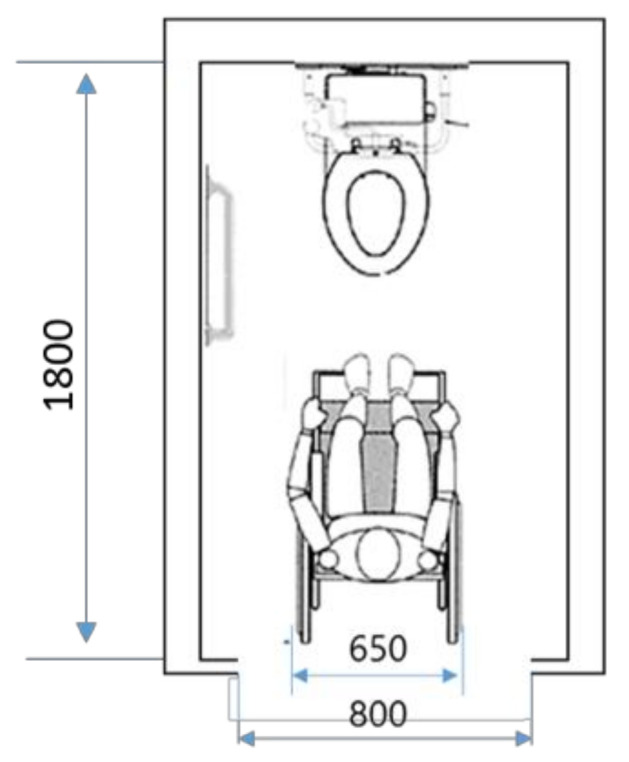
Size of wheelchair-compatible toilet [27] (Unit: mm).

**Figure 2:**
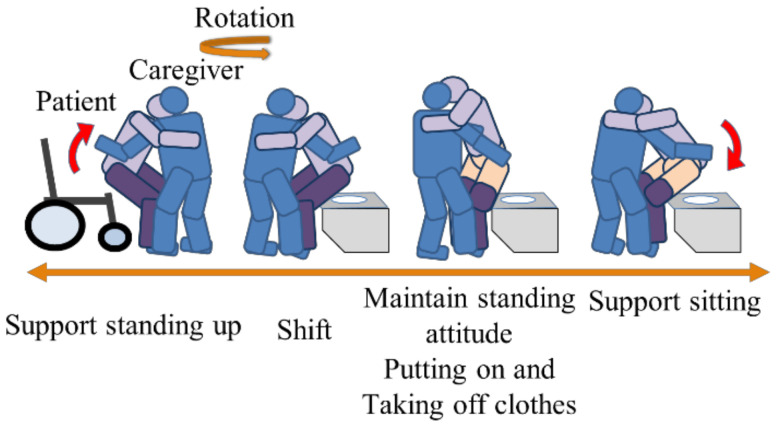
Transferring in toilet facilities for the elderly.

The toilet used on a daily basis was a wheelchair-adapted toilet [26] (Figure 1). This toilet has a width of about 1000 mm and a depth of about 1800 mm, and the maximum width of the wheelchair in the JIS standard is 700 mm [27], so the workspace of a caregiver is narrow. There were six facilities with multipurpose toilets with a wide workspace (width 1800 mm, depth 1800 mm), but they were not used on a daily basis because they were located away from the living room. Therefore, when performing a transfer operation in a wheelchair-compatible toilet, the space was severely restricted. Therefore, the transfer operation was performed not by two people but was carried out by one person using the front holding method as shown in Figure 2.

During the movement of transferring the elderly to the toilet, in order to prevent knee breakage due to a decline in their lower leg function, the caregiver fixes their knees, supports them from a wheelchair, moves to the toilet seat through a rotating motion, helps them put on or take off their clothes while holding their trunk with the upper arm, and supports them sitting. As an opinion regarding the transfer operation to the toilet, the rotational motions have a high risk of falling, and many respondents said that it was dangerous.

In all facilities, there was no toilet device other than handrails. Reasons for not using the transfer assistive device were mentioned as “if the elderly’s cognitive function is low, they will be surprised at the operation of the device”. It “cannot be used when upper limb/trunk is disabled”, or that they “would like to respond to caregiver dysfunctions and sudden movements by performing it manually”.

Therefore, in this study, the assisted transfer assisted is intended for a front holding method by one caregiver who can operate it even if the movement space is limited. As device restrictions, “the elderly can sit down”, “size applicable to wheelchair-compatible toilet”, and the device requirements for using within facilities include “elimination of rotational movement”, “operation by human hand”, “operation do not compress the upper limbs and upper torso.

### 2.3. Lumbar Burden in the Transfer Movement

As a direct method for measuring the compression force of the intervertebral disc (lumbar burden), there is a method of inserting an electrode into the intervertebral disc, but it is not a general method due to its high invasiveness. Therefore, as a non-invasive method for estimating the lumbar burden, a method for estimating the motion describing the motion of the human body as a rigid body link model, is adopted. Since the forces and moments applied to each segment obtained from the model analysis are balanced by the sum of the moments exerted by the muscles and surrounding elastic tissues around the center of the joint, the human skeleton of the lumbar spine and back muscles are also in the lumbar intervertebral disc. The method is used to estimate the lumbar burden by estimating the muscle tension from the average dimensional distance in the above position and adding the load obtained from the model applied to the lumbar spine and the load due to the muscle tension. It is a method to describe the movement by regarding each segment as a rigid body link mechanically connected by a ball joint [28]. Evaluation of the joint position when using a rigid body link of the human body has been promoted. In order to describe the motion of the rigid link model as an equation of motion and calculate the force acting on each link, it is necessary to measure the trajectory of the motion and the external force at the boundary surface. Therefore, in this study, we decided to measure the motion using a three-dimensional motion analysis device system (CORTEX3.6 manufactured by Motion Analysis) and a floor reaction force measurement device (AMTI).

The evaluation standard value of lumbar burden was 3400 N [29] set by NIOSH. In motion analysis, the human body was converted into a rigid link model, and the lumbar burden was evaluated with reference to Equation (1) of Yamazaki et al. Yamazaki [28] considered that the moment of the hip joint is generated by the surrounding muscle tension, estimated the total load due to muscle tension by dividing the moment by each muscle group and the moment arm at the center of the hip joint, and estimated their own weight. The lumbar disc compression force is calculated by adding the force of the component parallel to the lumbar spine and the external force.

(1) $$\sum F_{LC}=20M_{B}+8M_{L}+23M_{R}+F_{waist}$$

$M_{B},\;M_{L},\;M_{R},\;F_{waist}$ represents the forward bending moment, side bending moment, turning moment, self-weight component, and external force component applied to the center of the lumbar joint.

The rigid link model used to be a model in which the human body was divided into nine parts: the head, trunk, upper limbs, waist, both thighs, both lower legs, and both legs. In addition, as a feature of the caregiver’s standing support movement, it was assumed that the elbow joint angle of the assistant did not change greatly during the movement because the upper limb was not moved. Consider a link that integrates the trunk and upper limbs. The estimated body weight and moment of inertia of the human body and body length were calculated by [30,31].

#### 2.3.1. Grasping the Time When the Lumbar Burden Increases

The factors of lower back pain were analyzed according to the front holding method. Through this analysis, we can grasp the quantitative burden of the transfer caregiving action on the waist and the action and burden of other problems. By evaluating the actual actions of caregivers, it is considered that we can understand how to correspond to the characteristic diseases of the elderly being cared for.

The subjects were four male caregivers with no back pain disease D1–D4 (three physical therapists and one social worker: height 1.76 ± 0.03 m, weight 69.8 ± 2.9 kg) and two students E1, E2 (height 1.72 ± 0.03 m, weight 65.0 ± 4.3 kg).

**Figure 3:**
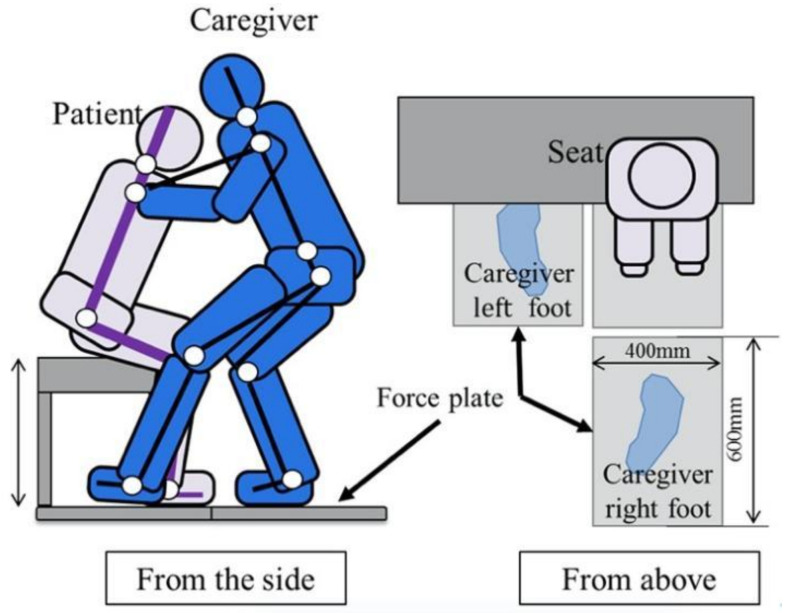
Humans’ position of the transferring operation.

E1, E2 sitting on the simple bed up to 50cm would be assisted by the caregiver through the front holding method in the series of actions of standing support (Sit-Stand), standing position maintenance (Hold) and seating support (Stand-Sit). Caregivers paid attention that the lower limbs do not touch the E1,E2. The sole of the foot is usually placed on a floor reaction force meter, and the contact of a floor reaction force meter is one point. E1 and E2 should imitate the complete weakness of the elderly. The experiment was performed with the positional relationship shown in Figure 3.

#### 2.3.2. Three-Dimensional Motion Analysis Device

As a means of measuring and analyzing the above burden evaluation indicators, we used the three-dimensional motion analysis device system (CORTEX3.6) and floor reaction force measurement device (manufactured by AMTI) produced by NAC for measurement. An infrared reflective marker was used, and the marker set was Helen Hayes Hospital Marker Set [32].

**Figure 4:**
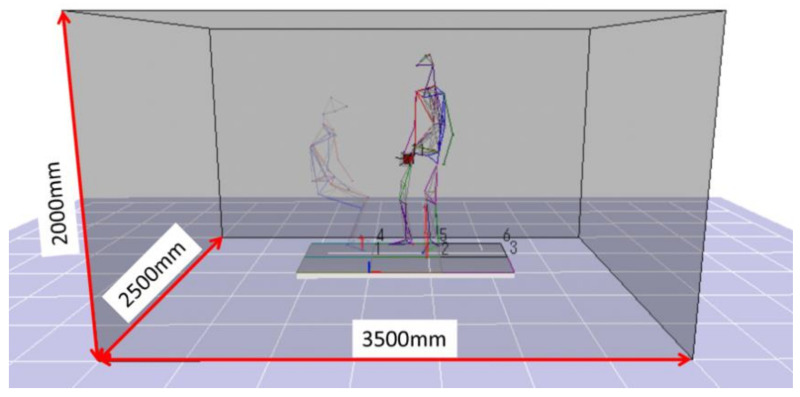
Movement measurement area.

**Figure 5:**
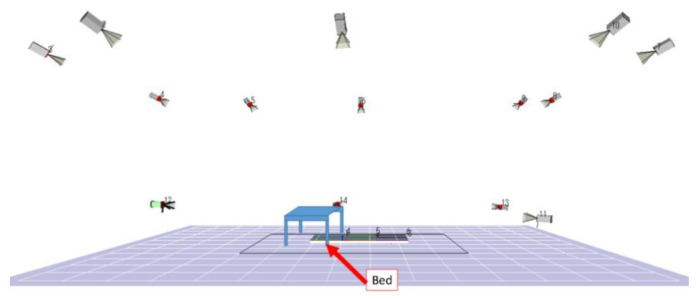
Side view of camera configuration during experiment.

In the experiment of this study, all the motion measurement experiments were carried out in the motion measurement room of the Institute of Gerontology (IOG) in the Kashiwa Campus of the University of Tokyo. The measurement conditions are 50 Hz. As shown in Figure 4, the movement measurement area of the room is 3500 mm wide, 250 mm deep, and 2000 mm high, which is enough to evaluate the transfer movement and preparatory research movement as the protagonist in this study. In addition, by synchronizing with the floor reaction force meter, the position and vector of the force on the floor reaction force can be measured according to the force and moment vector working on the floor reaction force. The camera configuration during the experiment is shown in Figure 5.

#### 2.3.3. Experimental Results and Discussion

**Figure 6:**
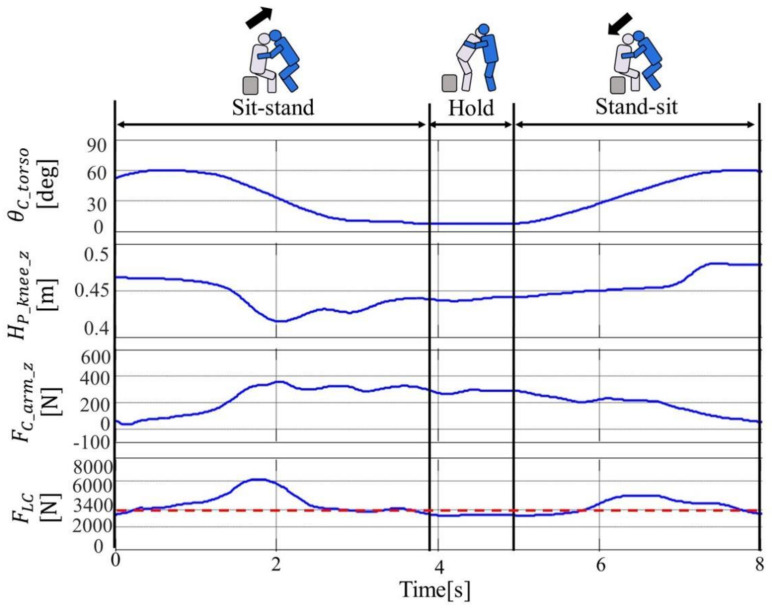
Time series response of D1 Caregiver in transferring load.

By analyzing the data collected by the three-dimensional motion analysis device, the time series response of subject D1 for trunk tilt angle $\;\theta_{C\_torso}$, knee joint height of the elderly $H_{P\_knee\_z}$, caregiver’s upper limb exertion $F_{C\_arm\_z}$, the lumbar disc compression force $F_{LC}$ are shown in Figure 6. Over most of the movement section,$\;F_{LC}$ exceeded the evaluation standard value of 3400 N, and the lumbar burden was large. Focusing on Sit-Stand, $H_{P\_knee\_z}$ decreased rapidly around 2 s, indicating the occurrence of knee bending in the elderly.$\;F_{C\_arm\_z}$ and $F_{LC}$ also increased at the same time as the elderly’s knee break, and $F_{LC}$ exceeded the evaluation standard value of 3400 N. Therefore, in Sit-Stand, the obtained result shows that the lumbar burden increased due to the knee of the elderly.

**Figure 7:**
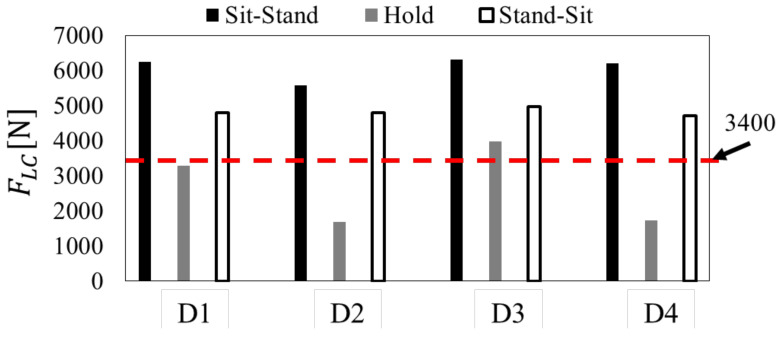
Maximum of $F_{LC}$ in each transferring movement.

Figure 7 shows the maximum value of $F_{LC}$ for each subject’s motion section. $F_{LC}$ exceeded 3400 N in Sit-Stand and Stand-Sit in all subjects, and $F_{LC}$ was around 3400 N in Hold. In addition, when the values for each section are compared, $F_{LC}$ is maximized in Sit-Stand.

The lumbar burden of the caregiver in the transfer movement is grasped, $F_{LC}$ increases with the increase of $F_{C\_arm\_z}\;$. In Sit-Stand, $\;F_{C\_arm\_z}$ was increased as a result of the knee break of the elderly. From these results, we extracted “weight compensation for the elderly to reduce $\;F_{C\_arm\_z}\;$” and “prevention of knee breakage of the elderly” as the device requirements for reducing the burden on the waist of the caregiver.

### 2.4. Assistive Method Based on Motion Analysis

By measuring the Sit-Stand, Hold, and Stand-Sit when the knee joint of the elderly is fixed, it is verified that the burden of the caregiver and the elderly can be reduced when only the knee joint of the elderly is fixed. It will be determined whether this would become one of the requirements for device development.

The NAC three-dimensional motion analysis device described in Section 2.3.2, the simple bed (height 500 mm), and the mock-up described in the next are used as needed.

#### 2.4.1. Mock-Up

**Figure 8:**
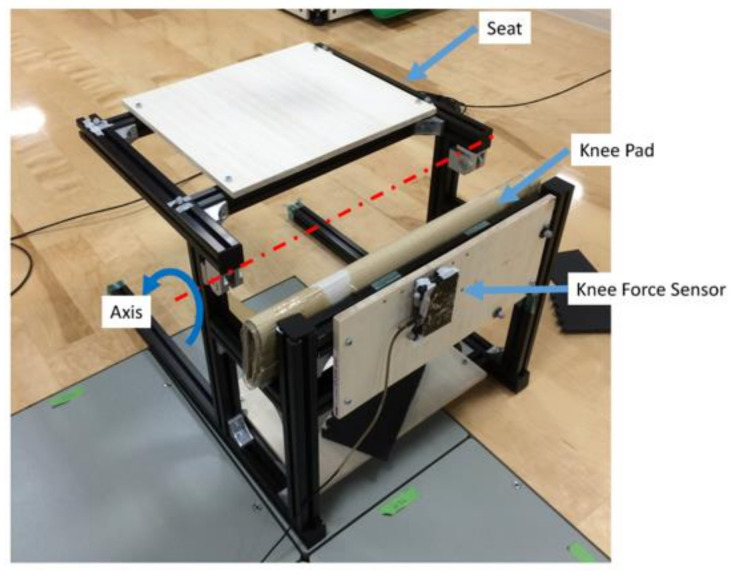
Appearance of the mock-up.

We used Misumi aluminum frame to make a mock-up (Figure 8) that can fix the knee joint of the elderly and lift the seat surface. This mock-up is also loaded with a pedal dynamometer (force sensor) of Kyowa Electronic Instruments Co., Ltd. (Chofu City, Japan) with a rated capacity of 500 N. It can measure the reaction force generated by the contact between the caregiver’s knee joint and the mock-up, and the experiment can be carried out in synchronization with the three-dimensional motion analysis device.

#### 2.4.2. Movement Assisted by Device

**Figure 9:**
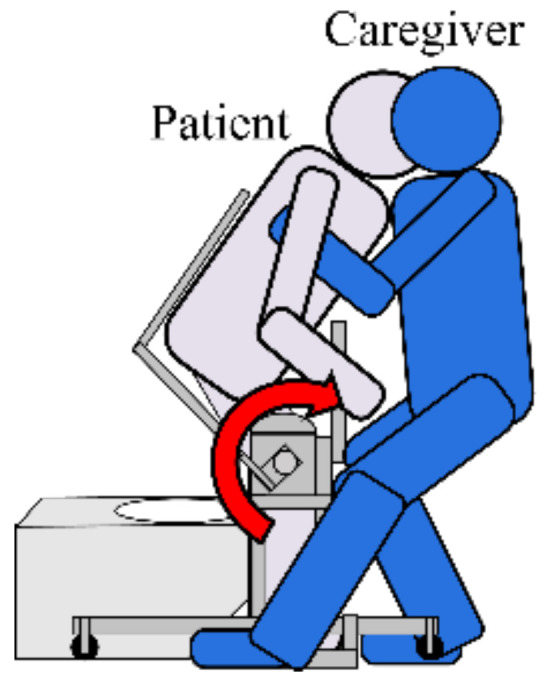
Conceptual diagram of the device in use.

Based on the results obtained in Section 2.3.3, the movement with a large lumbar burden was the Sit-Stand. We considered that the device supports the Sit-Stand as shown in Figure 9.

In the front holding method, when the lumbar burden factor (the leaning angle of the caregiver) increases, the lumbar burden also increases. For this reason, the method in which the caregiver grasps the elderly does not increase the trunk forward tilt angle, as shown in Figure 9, and considers the method of holding the elderly with both hands as the target of the assistive movement.

#### 2.4.3. Lumbar Burden Factor of Caregiver

Since the transfer motion of the caregiver while using the front holding method is a two-dimensional [29] movement, the dominant term in Equation (1) is the forward bending moment on the waist of the caregiver. Equation (2) shows the equation for calculating the forward bending moment.

(2) $$\;\;\;\;\;\;\;\;\;\;\;\;\;\;\;M_{B}≅{\vec{F}}_{C\_arm}*{\vec{r}}_{C\_arm}+W_{C\_upbody}*L_{C\_upbody}sin\theta_{C\_torso}$$

However, ${\vec{F}}_{C\_arm}$ is the caregiver’s upper limb exertion force, ${\vec{r}}_{C\_arm}$ is the moment arm (vector from the waist joint to the grip), $W_{C\_upbody}$ is the weight of the caregiver’s head and upper torso, $L_{C\_upbody}$ is the length of the body, including the upper torso and head of the caregiver, and $\theta_{C\_torso}$ is the leaning angle of the caregiver. Since the transfer operation is a relatively slow operation, we exclude the dynamic term. In this Equation (2), the terms that change during the movement are the trunk tilt angle of the caregiver $\theta_{C\_torso}$, the upper limb exertion power of the caregiver ${\vec{F}}_{C\_arm}$, and the moment arm ${\vec{r}}_{C\_arm}$. Therefore, these three variables were set as lumbar burden factors.

#### 2.4.4. Extraction of Lumbar Burden Factor in Transfer Movement

Under the condition of knee fixation of the elderly being assisted, the timing at which the lumbar burden of the caregiver increases during the Sit-Stand and the lumbar burden factor are extracted.

**Figure 10:**
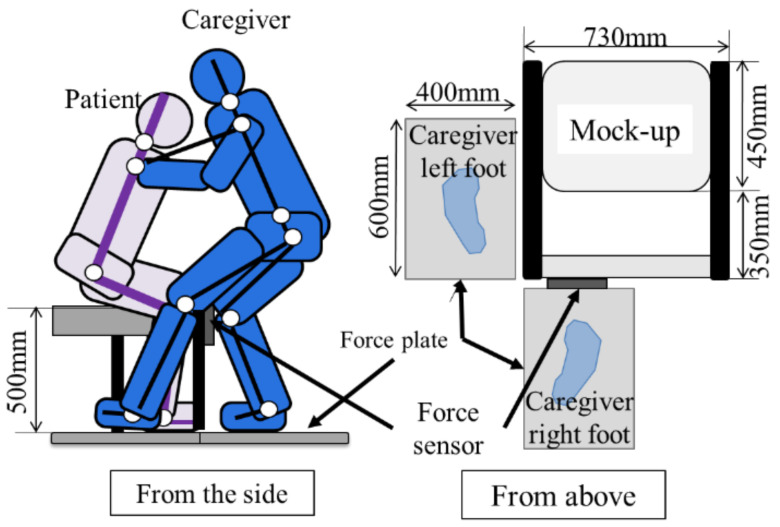
Situation of experiment: position of subjects and mock-up.

The subjects, experimental procedure and experimental devices used are the same as those in Section 2.3.1, and the mock-up in Section 2.4.1 is added, performed with the positional relationship shown in Figure 10. For the movement, the caregiver performed a Sit-Stand assistive movement using the front holding method to the elderly, and the state was measured.

#### 2.4.5. Experimental Results and Discussion

**Figure 11:**
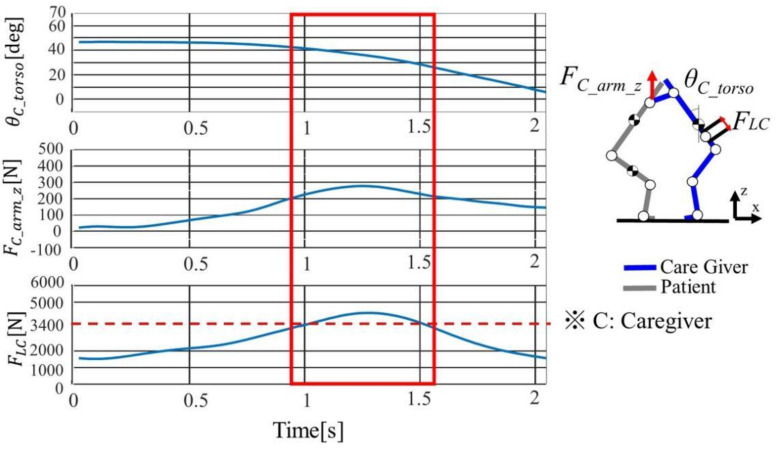
Caregiver’s load for assisting elderly stand up when the knees were fixed (D1).

The operation was from the time when the caregiver started to raise the trunk to the time when the erecting was performed, and the average of the operating time was 2.76 ± 0.30 s. Figure 11 shown the time series responses of the caregiver’s trunk tilt angle $\theta_{C\_torso}$, upper limb exertion force $F_{C\_arm_{z\;}}$, lumbar disc compression force $F_{LC}$ during the Sit-Stand of a representative subject D1 (height: 1.72 m, weight: 71.0 kg). As for Sit-Stand movement, $\theta_{C\_torso}$ decreases with time. There was a tendency for $F_{C\_arm\_z}$ to increase when $F_{LC}$ increased. In addition, at the time surrounded by red squares, the evaluation standard value of 3400 N was exceeded, indicating that Sit-Stand movement is with a high risk of back pain.

**Figure 12:**
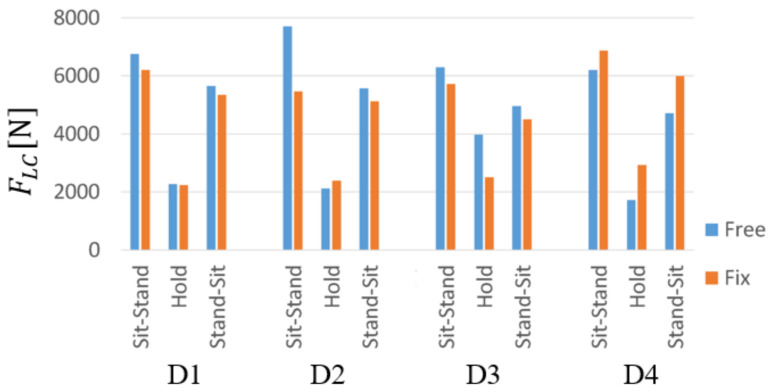
$F_{LC}$ in each transferring movement (Free: Unfixed knee, Fix: Fixed knee).

In addition, each movement is evaluated separately. As in Section 2.3.3, the maximum value was drawn for the Sit-Stand and Stand-Sit, and the average value was drawn for the holding sections. The comparison of disc compression is shown in Figure 12.

In Sit-Stand, the difference between unfixed and fixed knees in D1 and D3 is about 500 N. For D2, a difference in the decrease of $F_{LC}$ of 2500 N was observed. It was also confirmed that $F_{LC}$ in Hold, all subjects were less than 3400 N. Only in D4 did the caregiver’s $F_{LC}$ increase.

According to the subjective ideas of caregivers, it would be easier to carry out standing assistance with the fixation of knee joints. D3 reduces the burden of movement and can carry out caregiving more safely than usual. On the other hand, the sense of stability of D1 and D2 increased compared with that fixing the knee by themselves. However, if they fix the knee by themselves, they could judge whether the lower limb muscle strength of the elderly is unstable according to the reaction force of the elderly, but it may be difficult to judge if the knee is fixed mechanically.

In all subjects, it is confirmed that the average value of the lumbar disc compression force in the standing maintenance interval is lower than 3400 N. Therefore, in this study, it can be said that the lumbar burden of the caregiver and the upper limb burden of the elderly are fully reduced in terms of seat lifting support during standing and sitting, and knee joint fixation during standing hold.

## 3. Development of Transfer Assistive Device

Based on on-site observation and working characteristics of the facilities for the elderly, the development conditions of the device are summarized, and the contact between the caregiver and the elderly is regarded as the restrictive condition for the development. We have grasped the problem points in the transfer movement, determined the movement outline of the device based on this, and grasped its necessity and effect. In this section, the specifications of the device are determined in detail.

### 3.1. Functional Requirements

- Standing/sitting motion assistance

To reduce the load on the upper limbs of the caregiver and the elderly, torque can be generated around the knee joint of the elderly. In order to allow the elderly’s weight to reach 80 kg and the rated torque to be 250 nm, the maximum angular speed is 30 deg/s according to the speed requirements of transfer movement.

- Standing hold assistance

In order to improve the stability of the lower limbs of the elderly, the lower limbs of the elderly can be mechanically fixed.

- Mobility assistance

Considering the movement between seat surfaces in sitting posture, it should have a backrest, wheel, and operating handle.

- Seat attachment/detachment

It has a shape that can be laid under the buttocks of the elderly at all times and can be easily attached and detached when the assistive device approaches.

### 3.2. Device Specifications

**Figure 13:**
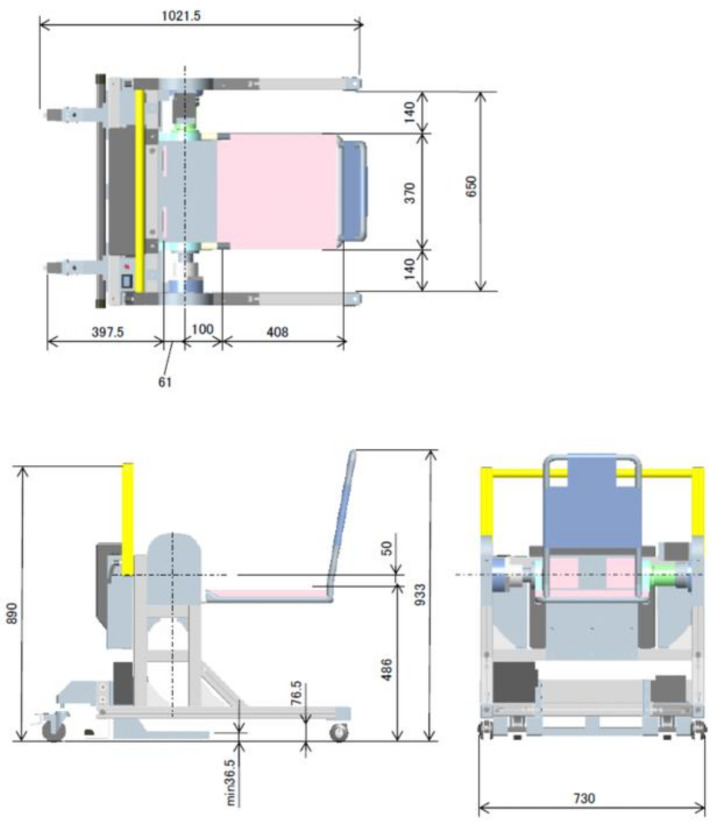
Dimension overview of the proposed device (Unit: mm).

**Figure 14:**
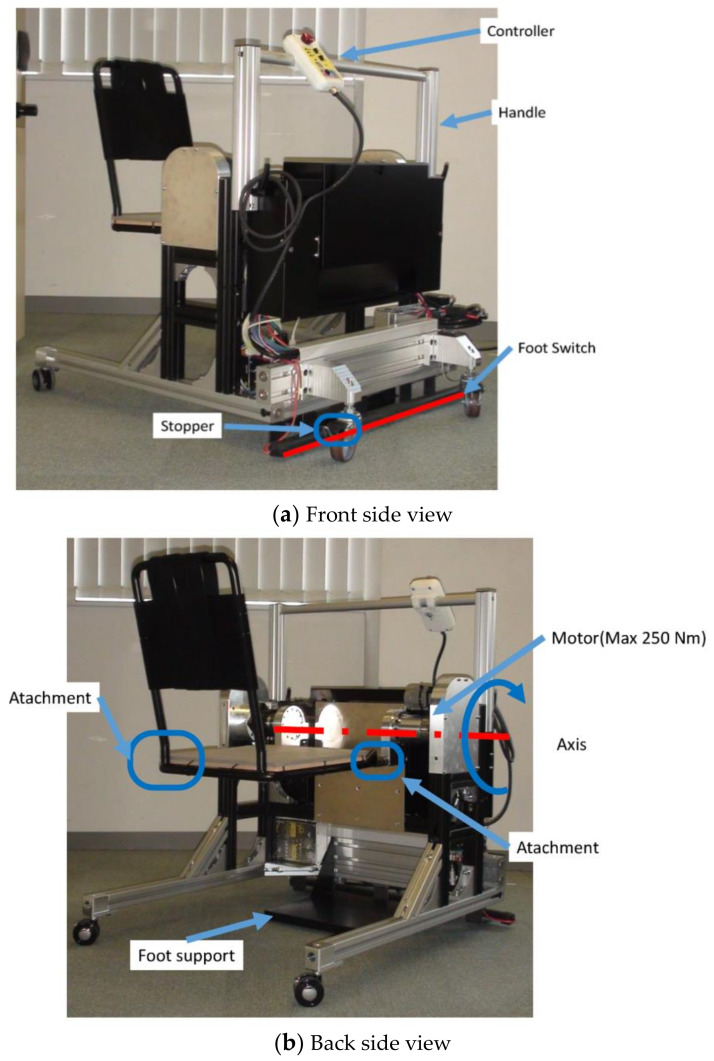
The proposed device appearance.

The proposed mechanism satisfies the constraints and device requirements aforementioned. Table 2 summarizes the mechanisms corresponding to the device requirements and constraints extracted in Section 2, Figure 13 and Figure 14 show the dimension overview and the appearance of the created device mechanisms.

As a feature, it has a knee-fixing device to prevent knee breakage for the elderly, and to enter the toilet with the elderly on the transfer assistive device in order to eliminate rotational movement in the toilet. It is possible to move the elderly on the toilet seat, and in order to compensate the weight of the elderly, torque is applied around the knee joint of the elderly. This mechanism can assist the elderly from the thigh by raising and lowering the seat.

**Figure 15:**
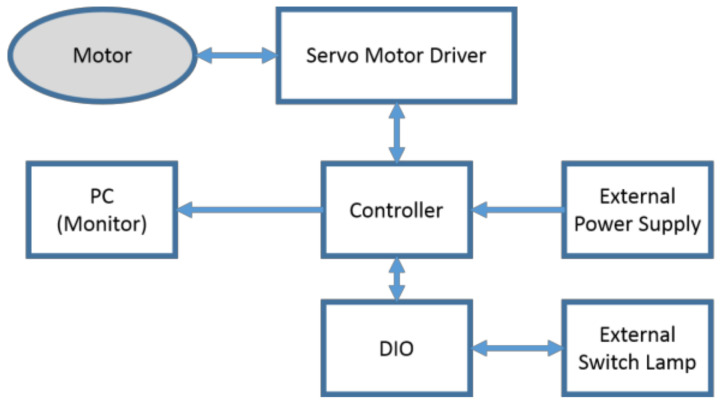
System configuration of the proposed device.

A servo motor is used for a controller to be the drive system [33] (Figure 15), with ratings of 200 W, 0.64 nm, and 6.4 A are set to 564.71 times using a wave gear speed reducer (harmonic drive) and a gear to obtain an output side rated torque of 253 nm to allow a person to be weighed up to 80 kg. The control program created on the PC is mounted by the servomotor controller internal program (Tamagawa Seiki Motion Designer), the motor position, rotation speed and output torque are acquired based on the current value and the encoder, and processed by the controller, the command is determined by current or pulse and controlled. In addition, information obtained from the controller is recorded by outputting it to the notebook computer R731 (TOSHIBA) using a USB serial cable. Power is supplied by Cosel’s DC 48 V power supply (ADA750F-48-R) used for servo motor (TS4607 N7001E620), controller (TA8440 N2300E100) and driver (TA8410 N3518E697). Due to motor characteristics, in the continuous region, one capable of exhibiting the rated torque in the range up to 3000 rpm was used. Since the reduction ratio is 564 times, the rated torque is compensated in the operation up to about 32 deg/s when evaluated by the seat face angle to the output side.

### 3.3. Usage Method and Scenario

**Figure 16:**
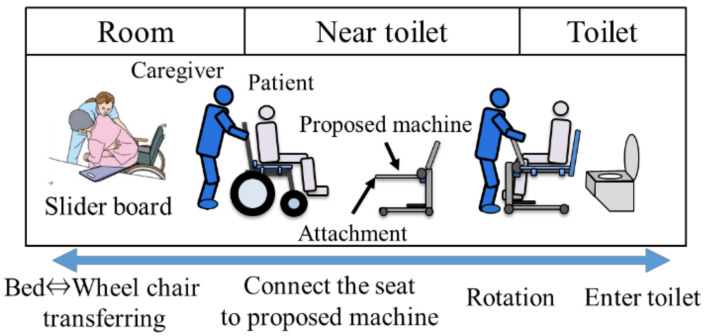
The way of using the proposed device from room to toilet.

**Figure 17:**
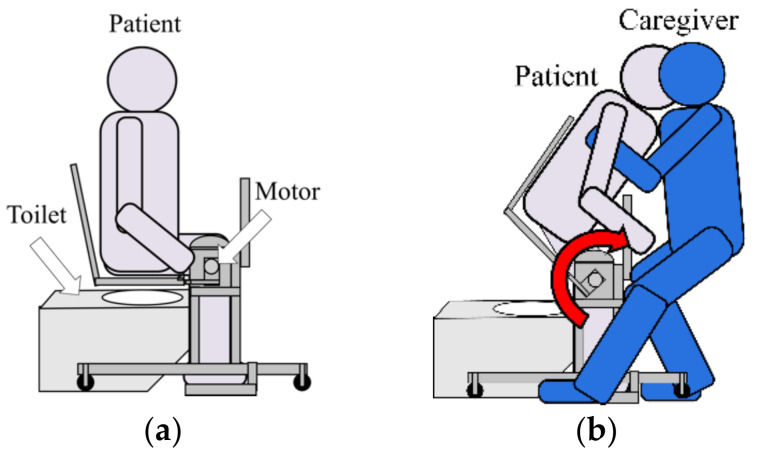
Two states of using the proposed device. (**a**) Sitting posture on the device; (**b**) Support standing up by using the device.

Figure 16 shown the operation of using the proposed device from the room to the toilet. First, a wheelchair is used as a means of transporting the elderly from the living room to the toilet. When the caregiver transfers the elderly to the wheelchair in the living room, a special chair is placed on the wheelchair seat and a slider board is used to transfer the person from the bed to the wheelchair. After moving from the room to the toilet vicinity and arriving near the toilet, the transfer assistive device placed near the toilet is attached to a dedicated chair. As for the mounting method, since pipes with a taper attach to both ends of the dedicated chair, the chair and the device are connected by inserting an attachment extending from the transfer assistive device into the hole of the pipe. At the same time, a special backrest is attached to the chair so that the person being assisted can be kept in the sitting position using only the transfer assistive device. Then, enter the toilet with the elderly sit on the transfer assistive device, and move the elderly onto the toilet seat. After that, torque is applied around the knee joint with a motor, and the state seating surface is raised and lowered from the state of Figure 17a, so that the elderly’s standing support and sitting support are provided as shown in Figure 17b. The device requirements shown in Table 2 have been achieved by this mechanism and usage [33].

## 4. Experiments: Operation Evaluation Using Transfer Assistive Device

### 4.1. Assistive Movement Evaluation

#### 4.1.1. Experimental Setup

In order to evaluate the appropriateness of the device, we use the designed transfer assistive device to evaluate the lumbar burden and the upper limb burden of the caregiver in the assistive movement.

In order to evaluate the burden of caregivers, we measured the lumbar disc compression as in Section 2, and performed the transfer movement in the same posture as in Figure 3 and Figure 10. When the caregiver pressed the knee joint, as in Section 2.4, the pedal dynamometer produced by Kyowa Electronic Instruments Co., Ltd. would be used for measurement. The three-dimensional motion analysis device system and the floor reaction force measurement device described in Section 2.3.2 still need to be used to collect data and calculate the burden on the waist and upper limbs.

**Figure 18:**
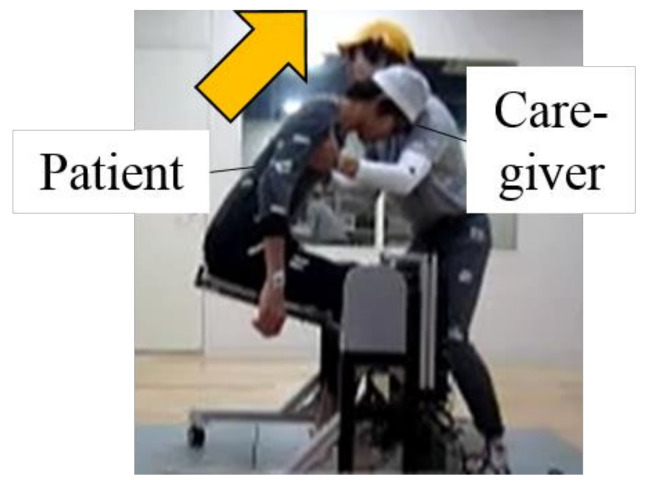
The appearance in the experiment.

The subjects in this experiment were 2 professional caregivers (male) D2, D3 and 4 students E1–E4. E1 and E2, E3 and E4 form a pair, and, alternately, perform actions in the roles of simulated caregiver and simulated elderly. The height, weight and professionalism were shown in Table 3. The appearance in the experiment is shown in the Figure 18.

#### 4.1.2. Experimental Method

In order to compare the effect of the proposed device, we designed to measure the transfer movement in the following three conditions. Since the voltage protection circuit of the driver is not installed in the power supply configuration of the proposed device, it is necessary to reduce the rapid reverse rotation of the motor caused by the load, so the Assist Rise only measured the rising movement.

- Control Condition

Without using the proposed transfer assistive device, the caregiver helps the elderly stand up or sit down from the seat.

- Automatic Lifting

The lifting or lowering operation is automatically initiated through the switch input of the proposed transfer assistive device.

- Assist Rise

The seat lifting or lowering operation of the elderly can be performed according to the intention of the caregiver. In other words, the caregiver can gently hold the upper body of the elderly to ensure their safety. At the same time, the seat can be raised or lowered.

#### 4.1.3. Experimental Results

Evaluations of the maximum value of lumbar force and upper limbs among subjects were performed. Knee keeping torque, which is a physique index of a simulated elderly by device, could be appropriately obtained except for E2 (simulated patient).

**Figure 19:**
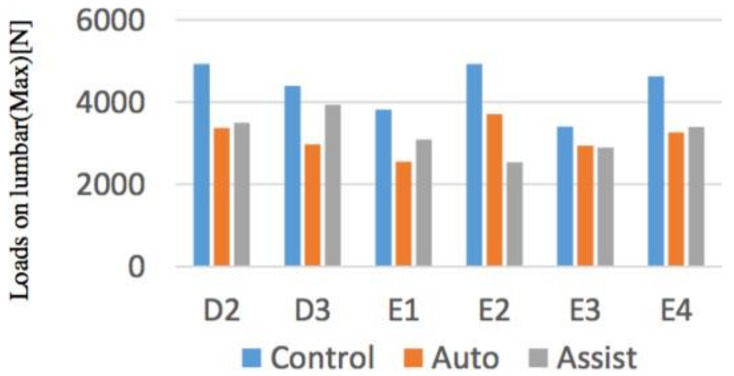
Loads on lumbar.

**Figure 20:**
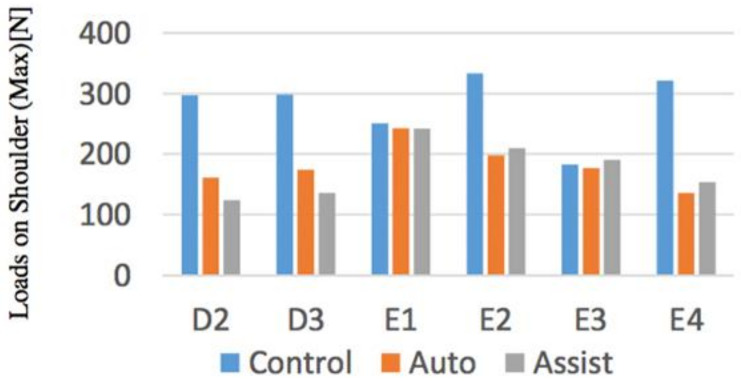
Loads on shoulder (upper limb).

The maximum value of the lumbar disc compression force between the subjects and the maximum value of the force applied to the upper limbs were evaluated. As objective results, from Figure 19 and Figure 20, by using the device in the movement other than D3, it was found that the loads on the waist returns to the safety standard. When comparing the forces applied to the upper limbs, the force decreased in operations other than E1 and E3. The reason why the change in the force applied to the upper limb in the operation of E1, E3 was small being that the loads on the Control Condition is small, so that it is thought that the simulated elderly E2, E4 in each operation were not sufficiently weak at the Control Condition.

#### 4.1.4. Discussion

From the above results, we found that when using the transfer assistive device, with the exception of D3, the rest of the subjects’ lumbar load reached the safety standard under the condition of Assist Rise. Therefore, we re-experimented D3 and conducted a more detailed analysis.

**Figure 21:**
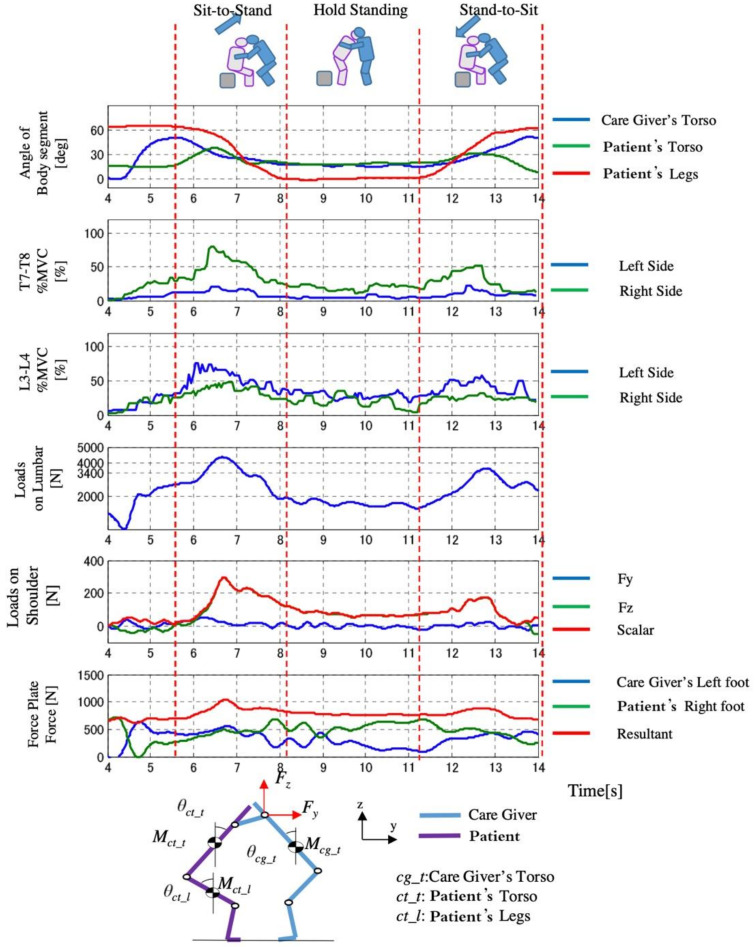
Change of loads with time in Control Condition (D3).

**Figure 22:**
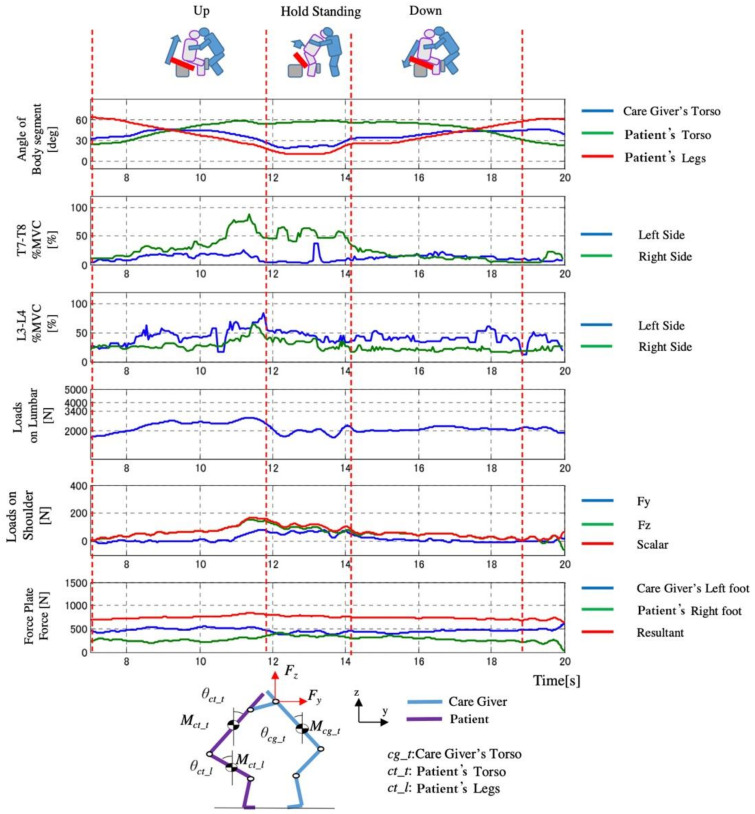
Change of loads with time in Auto Lifting (D3).

**Figure 23:**
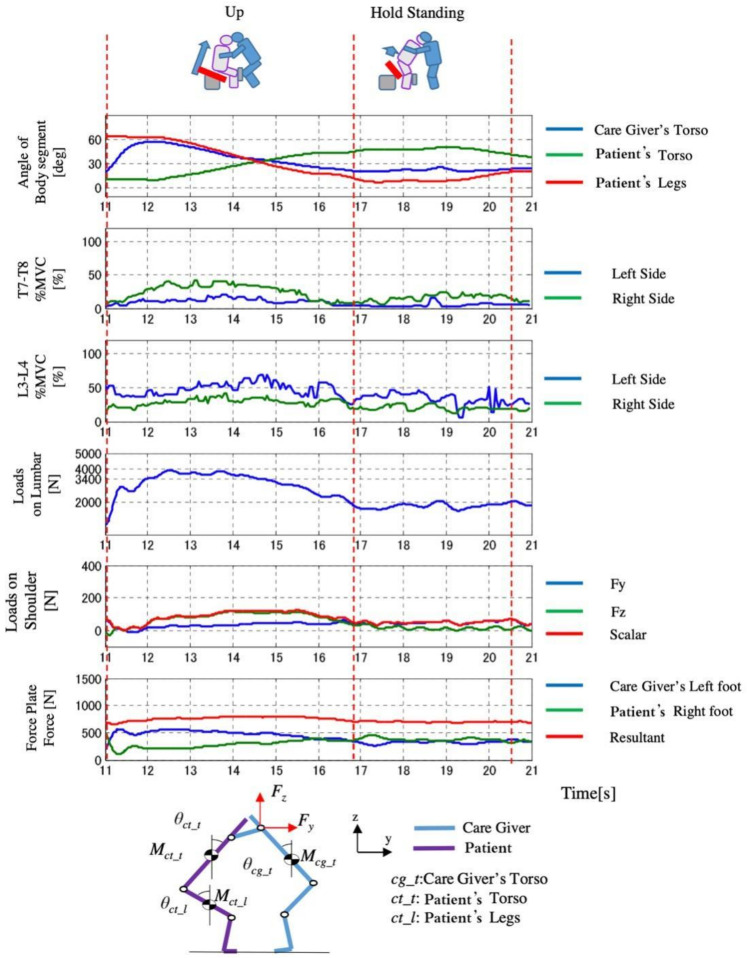
Change of loads with time in Assist Rise (D3).

The measured results of D3 are shown in Figure 21, Figure 22 and Figure 23. We found that in Control Condition (Figure 21), when the knee break occurs in the elderly, the lumbar loads, and the pressure of upper limb of the caregiver are the largest.

In the comparison of the load of conditions between Automatic Lifting and the Assist Rise, the pressure of the waist was almost the same as the safety standard. The loads of the intervertebral disk decreased in the Automatic Lifting. In the case of the Automatic lifting, since the operation of the caregiver is in contact with the patient (elderly) in the vicinity of 9 s (Figure 22), that is, the seat surface angle begins to rise, in Assist Rise, the caregiver starts forcibly by 12 s and 14 s in Figure 23, and the operation starts by supporting the elderly. It is because the assistance of the caregiver is carried out with a large load.

Regarding the load applied to the upper limbs of the caregiver, when Assist Rise and the Control Condition were compared, it decreased by 180 N, and the maximum value could be reduced to about 120 N. In Automatic Lifting, the maximum load applied to the upper limbs when getting out of seat surface after the seat movement stopped. There was no tendency of the change in the maximum value and a significant decreased tendency in the lumbar muscle presentation of the caregivers. During the D3 rise motion, the muscle exertion of the scoliotic muscle elevation position of the caregiver was the lowest in the device operation of the assist condition.

About the subjective opinion of D3, the following was obtained:

- Comparison of both conditions

1. There is no significant difference between the burden and the working time.

2. Since the stand-up allowance of the disengaged object who needs all-round help, it is best to decide the action according to the choice of the caregiver.

3. Assist operation is effective to prevent the wasting syndrome by using residual function.

- Assist operation

1. It is better to have a low initial speed and maximum speed in order to match the movements of the elderly and not to make sudden movements.

2. It is suitable for elderly people who have residual lower limb function, but cannot stand up sufficiently. It is desirable that the amount of assist can be adjusted in order to fully demonstrate the ability of the person being assisted.

- Seat raising and lowering operation in general

1. Since the seating surface is located near the buttocks when standing, the person being assisted can sit comfortably.

2. Because knee breaks do not occur, assistance can be provided with peace of mind.

3. There is a possibility that it can be applied to home assistance for elderly people who cannot get up from a wheelchair.

### 4.2. Operation in the Toilet

#### 4.2.1. Experimental Setup

Verification is carried out in practical applications and actual scenarios. To confirm the operation of the designed transfer assistive device, including moving it from the room to the toilet, and to extract by hearing the issues regarding the introduction of the designed assistive device by the caregiver of a series of actions in using the assistive device.

The experiment was conducted from the motion measurement room of the Institute of Gerontology (IOG) of the University of Tokyo to the multifunctional toilet on the floor.

In the multipurpose toilet, let D3 confirm the movement of the person being assisted from the wheelchair to the assistive device and the movement of the assistive device to enter the toilet.

#### 4.2.2. Experimental Results

**Figure 24:**
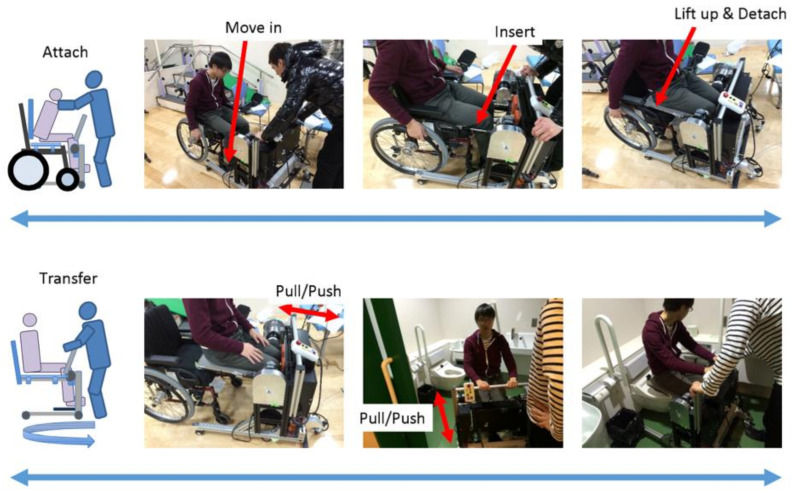
Operation overview in the toilet.

Figure 24 showed the outline of a series of actions by the caregiver.

We have only verified qualitatively, the developed transfer assistive device can be used in the actual multi-functional toilet, so there is no objective and specific data to explain it.

However, for the subjective evaluation results of caregivers, we summarized the opinion from D3.

- It is useful for transferring to the toilet of elderly people with reduced sitting abilities, which was difficult to transfer to the toilet, and can be used for many elderly residents.
- Elderly people who have begun to lose their standing ability due to the low possibility of knee breaks and backward falls will be able to go to the bathroom with peace of mind with almost independent movements.
- By adjusting the operation method and the strength of assistance, according to the caregiver and the elderly, the burden on the caregiver can be reduced, the independence of the elderly can be maintained. It is also useful for maintaining ADL for a long time from the viewpoint of disease prevention.

#### 4.2.3. Discussion

Based on the developed transfer assistive device can be used in the actual environment and the subjective ideas of caregivers. We also have the following discussion.

- Steering sits difficult with the elderly on the device, and it is necessary to simplify the approaching motion to the toilet and wheelchair.
- When transferring the elderly who has a low sitting hold ability and an unstable trunk, arm support during movement is required.
- It is necessary to consider the method of attaching and detaching the seat surface. In addition, the seat surface must be a soft component because it does not interfere with wheelchair seating (such as the use of cushions suitable for the elderly).

## 5. Conclusions

In this study, we proposed a transfer assistive device that can be applied to the elderly in facilities for those with upper limb upper body dysfunction and that meets the needs of on-site facilities to reduce the lumbar burden of caregivers when transfer movement occurred. This device focused on transferring the elderly from the room to the toilet in an integrated manner and allowing the elderly to excrete autonomously in the toilet. In addition, it can avoid lower back pain caused by the caregivers using the front holding method to support the elderly stand up, stand holding, sit down during the transfer process, as well as the fall caused by rotation. Based on the fixation of the knee joint and the comparison of three conditions, experiments have verified the effectiveness of the transfer assistive device, which reduced the caregiver’s lumbar burden within the standard value (3400 N) or lower. Furthermore, in the actual environment (room to toilet), a series of actions were verified in the actual application of the device.

The experimental results also shown that due to differences in personal habits or techniques, some caregivers inevitably exert pressure on the upper limbs and upper torso of the elderly during the transfer, and even produce the opposite effect. For these caregivers, it is important to unify the transfer assistive movements in advance.

As a future work, since this study only verified the standing assistive movement part, it is necessary to verify the sitting assistive movement part and design the control algorithm for the whole system to better meet the relationship of human-machine coordination. In addition, a large number of professional caregivers are needed to conduct the experiment as the subjects and fix the experimental subjects to conduct the experiment under different conditions, so as to make this study more rigorous as a strong control. On the other hand, the majority of caregivers (74.5%) who actually work in elderly facilities are women. Based on the differences between men and women, physical strength, body type, and more in line with the situation of elderly facilities, it is necessary to investigate whether the current lumbar burden reduction method is suitable for women, because the current subjects were men.

## Figures and Tables

**Table 1:** The situation of care service facilities which were investigated.

ID	Average Nursing Care Level	Numbers of Elderly	Numbers of Caregivers in the Daytime	Ration of Caregivers Having a Backache (%)	Number of Transfers for One Elderly Person (1 Day)
A1	4.1	60	14	Not clear	8
A2	3.9	60	8	30	16
A3	3.9	80	6.5	Not clear	8
A4	4.1	220	30	Not clear	8
A5	4.3	60	8	20	8
A6	4	38	4	80	18
A7	3.9	100	12	Not clear	8
B1	4.1	96	12	55	8
C1	4.1	140	Not clear	70	More than 8

**Table 2:** The requirements and device mechanisms.

Requirements	Mechanisms
Can be used in toilet	Proper size (width 730 mm)
Prevent rotation	Caster
Can be used for patients who have upper limbs or torso disorder	Caregivers can touch patients Assist from patient thighs
Prevent patients’ knee fold	Fix patients’ knee
Support patient’s weight	Assist of servo motor

**Table 3:** Subjects’ physique.

	D2	D3	E1	E2	E3	E4
Height (cm)	177	179	170	172	170	172
Weight (kg)	67	74	60	55	51	61
Qualification	PT	Social worker	Stu	Stu	Stu	Stu

## Data Availability

Data sharing not applicable.
